# Epigenetics and lupus

**DOI:** 10.1186/ar3935

**Published:** 2012-09-27

**Authors:** B Richardson

**Affiliations:** 1 University of Michigan, Ann Arbor, MI, USA

## 

Lupus develops when genetically predisposed people encounter environmental agents that initiate flares. Current evidence indicates that the environmental contribution is mediated by T-cell DNA demethylation. DNA methylation patterns are established during differentiation, and silence inappropriate or unnecessary genes by promoting a condensed chromatin configuration that is inaccessible to transcription factors. The methylation patterns are then replicated each time a cell divides by DNA methyltransferase 1 (Dnmt1). Dnmt1 is upregulated during mitosis, binds the replication fork, and catalyzes transfer of the methyl group from S-adenosylmethionine (SAM) to dC bases in the daughter DNA strand only where the parent strand is methylated. Environmental agents that block ERK pathway signaling prevent Dnmt1 upregulation, and low Dnmt1 levels synergize with dietary micronutrient deficiencies that decrease SAM pools to impair methylation of the daughter strand. This activates genes silenced only by DNA methylation.

Inhibiting T-cell DNA methylation converts helper CD4^+ ^T cells into autoreactive, cytotoxic, proinflammatory cells that cause lupus-like autoimmunity in mice. Similar changes in CD4^+ ^T-cell DNA methylation and gene expression are found in patients with active lupus. Procainamide and hydralazine, which cause ANAs in a majority of patients and lupus in a genetically predisposed subset, also inhibit T-cell DNA methylation. The lupus T-cell DNA methylation defect has been traced to low Dnmt1 levels caused by decreased ERK pathway signaling, and the signaling defect has now been traced to PKCδ inactivation caused by oxidative damage.

The importance of decreased ERK pathway signaling was confirmed by generating a transgenic mouse with an inducible dominant negative MEK. Inducing the signaling defect selectively in T cells decreases Dnmt1, causing anti-DNA antibodies in mice without lupus genes, and higher anti-DNA antibody levels and an immune complex glomerulonephritis in mice with lupus genes. Autoantibody levels and kidney disease are suppressed by dietary transmethylation micronutrient supplementation in these mice.

Epigenetic mechanisms also contribute to the gender dimorphism in lupus. Immune genes on the normally silenced X chromosome demethylate in women with active lupus, contributing to flare severity. In contrast, men with only one X chromosome require a greater genetic predisposition and/or greater degree of DNA demethylation to develop a lupus flare equal in severity to women.

Together, these studies indicate that environmental agents including oxidative stress and diet combine to inhibit T-cell DNA methylation, and that the epigenetically modified cells cause lupus-like autoimmunity in genetically predisposed people and mice.

